# Supplementary material for: The Difference in the Creativity of People Who Are Deaf or Hard of Hearing and Those with Typical Hearing: A Scoping Review
Source: Children (Basel). 2023 Aug 14;10(8):1383. doi: 10.3390/children10081383 (PMC10453058; doi:10.3390/children10081383)
Supplement: Supplementary file 1 [file children-10-01383-s001.zip › children-2552098-supplementary.pdf]

Supplementary Material. Search strategy

Table S1 Ovid MEDLINE(R) 1946

Search conducted on May 20, 2021

Search	Query	Results
1	„Creativity/“ [Mesh] OR „Imagination/“ [Mesh] OR „creativity“ [Ti/Ab/Key] OR „creative thinking“ [Ti/Ab/Key] OR „creative thinking abilit*“ [Ti/Ab/Key] OR “creative abilit*“ [Ti/Ab/Key] OR „abstract thinking*“ [Ti/Ab/Key] OR „creative activit*“ [Ti/Ab/Key] OR „creative training“ [Ti/Ab/Key] OR „imagination*“ [Ti/Ab/Key] OR „curiosity“ [Ti/Ab/Key] OR „originality“ [Ti/Ab/Key]	28,221
2	„Deafness/ OR Hearing Loss/“ [Mesh] OR „deafness“ [Ti/Ab/Key] OR „deaf“ [Ti/Ab/Key] OR „hearing loss“ [Ti/Ab/Key] OR “hearing impairment“ [Ti/Ab/Key] OR “hearing impaired“ [Ti/Ab/Key] OR „hearing disabilit*“ [Ti/Ab/Key] OR „hearing disorder*“ [Ti/Ab/Key] OR „hypoacus?s“ [Ti/Ab/Key] OR “defective hearing*“ [Ti/Ab/Key] OR „hard of hearing“ [Ti/Ab/Key]	82,496
3	#1 AND #2	89

Table S2 CINAHL Plus with Full Text

Search conducted on May 20, 2021

Search	Query	Results
1	„Creativness“ [Exact Subject Heading] OR „Imagination+“ [Exact Suject Heading] OR „creative thinking abilit*“ [Ti/Ab/Su] OR “creative abilit*“ [Ti/Ab/Su] OR „abstract thinking“ [Ti/Ab/Su] OR „creative activit*“ [Ti/Ab/Su] OR „creative training“ [Ti/Ab/Su] OR „imagination*“ [Ti/Ab/Su] OR „curiosity“ [Ti/Ab/Su] OR „originality“ [Ti/Ab/Su]	22,766
2	„Deaf-Blind Disorders“ [Exact Subject Heading] OR „deafness“ [Ti/Ab/Su] OR „deaf“ [Ti/Ab/Su] OR „hearing loss“ [Ti/Ab/Su] OR “hearing impairment“ [Ti/Ab/Su] OR “hearing impaired“ [Ti/Ab/Su] OR „hearing disabilit*“ [Ti/Ab/Su] OR „hearing disorder*“ [Ti/Ab/Su] OR „hypoacus?s“ [Ti/Ab/Su] OR “defective hearing*“ [Ti/Ab/Su] OR „hard of hearing“ [Ti/Ab/Su]	44,991
3	#1 AND #2	86

Table S3 ProQuest Central

Search conducted on May 20, 2021

Search	Query	Results
1	„Creativity“ [Ti/Ab/Su] OR „creative thinking“ [Ti/Ab/Su] OR „creative thinking abilit*“ [Ti/Ab/Su] OR “creative abilit*“ [Ti/Ab/Su] OR „abstract thinking“ [Ti/Ab/Su] OR „creative activit*“ [Ti/Ab/Su] OR „creative training“ [Ti/Ab/Su] OR „imagination“ [Ti/Ab/Su] OR „curiosity“ [Ti/Ab/Su] OR „originality“ [Ti/Ab/Su]	1,118,809
2	„Deafness“ [Ti/Ab/Su] OR „deaf“ [Ti/Ab/Su] OR „hearing loss“ [Ti/Ab/Su] OR “hearing impairment“ [Ti/Ab/Su] OR “hearing impaired“ [Ti/Ab/Su] OR „hearing disabilit*“ [Ti/Ab/Su] OR „hearing disorder*“ [Ti/Ab/Su] OR „hypoacus?s“ [Ti/Ab/Su] OR “defective hearing*“ [Ti/Ab/Su] OR „hard of hearing“ [Ti/Ab/Su]	144,615
3	#1 AND #2	603
4	Exclude: Newspapers, Wire feeds, Podcasts, websites, blogs, Trade journals, other journals, Artistic and aesthetics works	144

Table S4 EBM Reviews **(**ACP Journal Club (ACP), Cochrane Central Register of Controlled Trials (CCTR), Cochrane Database of Systematic Reviews (COCH), Cochrane Methodology Register Database (CMR), Database of Abstracts of Reviews of Effects (DARE), Health Technology Assessment Database (HTA), National Health Service Economic Evaluation Database (NHSEED)**)**

Search conducted on May 20, 2021

Search	Query	Results
1	„Creativity“ [Ti/Ab/Key] OR „creative thinking“ [Ti/Ab/Key] OR „creative thinking abilit*“ [Ti/Ab/Key] OR “creative abilit*“ [Ti/Ab/Key] OR „abstract thinking“ [Ti/Ab/Key] OR „creative activit*“ [Ti/Ab/Key] OR „creative training“ [Ti/Ab/Key] OR „imagination“ [Ti/Ab/Key] OR „curiosity“ [Ti/Ab/Key] OR „originality“ [Ti/Ab/Key]	951
2	„Deafness“ [Ti/Ab/Key] OR „deaf“ [Ti/Ab/Key] OR „hearing loss“ [Ti/Ab/Key] OR “hearing impairment“ [Ti/Ab/Key] OR “hearing impaired“ [Ti/Ab/Key] OR „hearing disabilit*“ [Ti/Ab/Key] OR „hearing disorder*“ [Ti/Ab/Key] OR „hypoacus?s“ [Ti/Ab/Key] OR “defective hearing*“ [Ti/Ab/Key] OR „hard of hearing“ [Ti/Ab/Key	4,300
3	#1 AND #2	5

Table S5 Annual Reviews

Search conducted on May 20, 2021

Search	Query	Results
1	Deaf [Ti/Ab/Key] OR deafness [Ti/Ab/Key] OR hearing loss [Ti/Ab/Key]	1
2	Creativitiy [Ti/Ab/Key] OR creativeness [Ti/Ab/Key] OR curiosity [Ti/Ab/Key]	6
3	#1 AND #2	0

Table S6 Clinical trials

Search conducted on May 20, 2021

Search	Query	Results
1	deaf OR deafness OR hearing loss OR hearing impairment OR hearing impaired OR hearing disability OR hearing disabilities OR hearing disorder OR hearing disorders) AND Other terms: (creativity OR creativeness OR creative thinking OR abstract thinking OR crreative ability OR creative abilities OR imagination OR curiosity OR originality	5

Table S7 Current controlled trials

Search conducted on May 20, 2021

Search	Query	Results
1	creativity OR creativeness OR creative thinking OR abstract thinking OR crreative ability OR creative abilities OR imagination OR curiosity OR originality) AND Condition: (deaf OR deafness OR hearing loss OR hearing impairment OR hearing impaired OR hearing disability OR hearing disabilities OR hearing disorder OR hearing disorders OR hard of hearing	0

Table S8 The Cochrane Central Register of Controlled Trials (CENTRAL - PubMed, EMBASE, CINAHL, Clinical trials a International Clinical Trials Registry Platform (IC-TRP))

Search conducted on May 20, 2021

Search	Query	Results
1	deaf OR deafness OR hearing loss OR hearing impairment OR hearing impaired OR hearing disability OR hearing disabilities OR hearing disorder OR hearing disorders) AND (emotion OR emotions OR emotional intelligence	196

Table S9 Web of Science Core Collection

Search conducted on May 21, 2021

Search	Query	Results
1	„Creativity“ [Ti/Ab] OR „creative thinking“ [Ti/Ab] OR „creative thinking abilit*“ [Ti/Ab] OR „creative abilit*“ [Ti/Ab] OR „abstract thinking“ [Ti/Ab] OR „creative activit*“ [Ti/Ab] OR „creative training“ [Ti/Ab] OR „imagination“ [Ti/Ab] OR „curiosity“ [Ti/Ab] OR „originality“ [Ti/Ab]	238,398
2	Deafness“ [Ti/Ab] OR „deaf“ [Ti/Ab] OR „hearing loss“ [Ti/Ab] OR „hearing impairment“ [Ti/Ab] OR „hearing impaired“ [Ti/Ab] OR „hearing disabilit*“ [Ti/Ab] OR „hearing disorder*“ [Ti/Ab] OR „hypoacus?s“ [Ti/Ab] OR „defective hearing*“ [Ti/Ab] OR „hard of hearing“ [Ti/Ab]	86,235
3	#1 AND #2	231

Table S10 Scopus

Search conducted on May 21, 2021

Search	Query	Results
1	„Creativity“ [Ti/Ab] OR „creative thinking“ [Ti/Ab] OR „creative thinking abilit*“ [Ti/Ab] OR „creative abilit*“ [Ti/Ab] OR „abstract thinking“ [Ti/Ab] OR „creative activit*“ [Ti/Ab] OR „creative training“ [Ti/Ab] OR „imagination“ [Ti/Ab] OR „curiosity“ [Ti/Ab] OR „originality“ [Ti/Ab]	314,782
2	„Deafness“ [Ti/Ab] OR „deaf“ [Ti/Ab] OR „hearing loss“ [Ti/Ab] OR „hearing impairment“ [Ti/Ab] OR „hearing impaired“ [Ti/Ab] OR „hearing disabilit*“ [Ti/Ab] OR „hearing disorder*“ [Ti/Ab] OR „hypoacus?s“ [Ti/Ab] OR „defective hearing*“ [Ti/Ab] OR „hard of hearing“ [Ti/Ab]	124,648
3	#1 AND #2	301

Table S11 PsycArticles

Search conducted on May 21, 2021

Search	Query	Results
1	„Creativity“ [MA MeSH Subject Heading] OR „Imagination“ [MA MeSH Subject Heading] OR „Creativity“ [Ti/Ab/KW] OR „creative thinking“ [Ti/Ab/KW] OR „creative thinking abilit*“ [Ti/Ab/KW] OR „creative abilit*“ [Ti/Ab/KW] OR „abstract thinking“ [Ti/Ab/KW] OR „creative activit*“ [Ti/Ab/KW] OR „creative training“ [Ti/Ab/KW] OR „imagination“ [Ti/Ab/KW] OR „curiosity“ [Ti/Ab/KW] OR „originality“ [Ti/Ab]	3,016
2	„Deafness“ [MA MeSH Subject Heading] OR „hearing loss“ [MA MeSH Subject Heading] OR „Deafness“ [Ti/Ab/KW] OR „deaf“ [Ti/Ab/KW] OR „hearing loss“ [Ti/Ab/KW] OR „hearing impairment“ [Ti/Ab/KW] OR „hearing im-	485

	paired“ [Ti/Ab/KW] OR „hearing disabilit*“ [Ti/Ab/KW] OR „hearing disorder*“ [Ti/Ab/KW] OR „hypoacusis“ [Ti/Ab/KW] OR „defective hearing*“ [Ti/Ab/KW] OR „hard of hearing“ [Ti/Ab/KW]	
3	#1 AND #2	7

Table S12 PsycInfo

Search conducted on May 21, 2021

Search	Query	Results
1	„Creativity“ [MA MeSH Subject Heading] OR „Imagination“ [MA MeSH Subject Heading] OR „Creativity“ [Ti/Ab/KW] OR „creative thinking“ [Ti/Ab/KW] OR „creative thinking abilit*“ [Ti/Ab/KW] OR „creative abilit*“ [Ti/Ab/KW] OR „abstract thinking“ [Ti/Ab/KW] OR „creative activit*“ [Ti/Ab/KW] OR „creative training“ [Ti/Ab/KW] OR „imagination“ [Ti/Ab/KW] OR „curiosity“ [Ti/Ab/KW] OR „originality“ [Ti/Ab]	80,294
2	„Deafness“ [MA MeSH Subject Heading] OR „hearing loss“ [MA MeSH Subject Heading] OR „Deafness“ [Ti/Ab/KW] OR „deaf“ [Ti/Ab/KW] OR „hearing loss“ [Ti/Ab/KW] OR „hearing impairment“ [Ti/Ab/KW] OR „hearing impaired“ [Ti/Ab/KW] OR „hearing disabilit*“ [Ti/Ab/KW] OR „hearing disorder*“ [Ti/Ab/KW] OR „hypoacus?s“ [Ti/Ab/KW] OR „defective hearing*“ [Ti/Ab/KW] OR „hard of hearing“ [Ti/Ab/KW]	29,849
3	#1 AND #2	192

Table S13 Google Scholar

Search conducted on May 24, 2021

Search	Query	Results
1	allintitle: ("creative" OR "creativity" OR "imagination" OR "imagine" OR "abstract thinking" OR "curiosity" OR "originality") AND ("hearing loss" OR "deafness" OR "deaf" OR "hearing impaired" OR "hearing impairment")	53
